# Measuring fidelity to delivery of a new smoking cessation intervention integrated into routine tuberculosis care in Pakistan and Bangladesh: Contextual differences and opportunities

**DOI:** 10.18332/tid/133054

**Published:** 2021-04-08

**Authors:** Melanie Boeckmann, Omara Dogar, Saima Saeed, Arman Majidulla, Shilpi Swami, Amina Khan, Kamran Siddiqi, Daniel Kotz

**Affiliations:** 1 School of Public Health, Bielefeld University, Bielefeld, Germany; 2 Department of Health Sciences, University of York, York, United Kingdom; 3 Addiction Research and Clinical Epidemiology Unit, Institute of General Practice, Medical Faculty of the Heinrich-Heine-University, Düsseldorf, Germany; 4 Usher Institute, University of Edinburgh, Edinburgh, United Kingdom; 5 Indus Hospital, Karachi City, Pakistan; 6 Evidera, London, United Kingdom; 7 The Initiative, Islamabad, Pakistan; 8 Hull York Medical School, University of York, York, United Kingdom

**Keywords:** tobacco, behavior change, South Asia, primary health care

## Abstract

**INTRODUCTION:**

Tobacco smoking among tuberculosis (TB) patients leads to poorer treatment outcomes. Smoking cessation support should be integrated into routine TB care. We measured healthcare providers’ fidelity to a smoking cessation intervention integrated into routine TB care, in Bangladesh and Pakistan. We aimed to understand the role of providers and settings in the implementation of behavior support (BS) messages for TB and smoking cessation.

**METHODS:**

The integrated BS intervention was implemented in TB clinics (24 public and 1 private). Cross-sectional data were collected on the fidelity of delivery of the BS intervention using a predefined fidelity index based on an existing validated method of measuring intervention fidelity. Audio-recordings of patient-provider BS sessions were coded using the fidelity index. Intervention fidelity was presented as the proportion of sessions that implemented BS messages.

**RESULTS:**

A total of 96 sessions were conducted, 37 in Bangladesh and 59 in Pakistan. In public settings, TB medication advice was offered in 91.9% (95% CI: 78.7– 97.2) of sessions in Bangladesh, and in 75.5% (95% CI: 62.4–85.1) of sessions in Pakistan; whilst it was offered in 83.3% (95% CI: 43.7–97.0) of sessions in the private setting in Pakistan. Patients’ smoking status was assessed in 70.3% (95% CI: 54.2–82.5) of sessions in Bangladesh, and in 34.0% (95% CI: 22.7–47.4) of sessions in the public setting and in 66.7% (95% CI: 30.0–90.3) of sessions in the private setting in Pakistan. A quit date was set in 32.4% (95% CI: 19.6–48.5) of all sessions in Bangladesh, and in 33.3% (95% CI: 9.6–70.0) of all sessions in the public setting in Pakistan.

**CONCLUSIONS:**

Fidelity to the intended delivery of the intervention was found to be high for TB-related messages but not for smoking cessation messages. Clinic contexts may play a mediating role in health workers’ opportunities to deliver the intervention as planned.

**TRIAL REGISTRATION:**

International Standard Randomized Clinical Trial Number (ISRCTN43811467). Registered 23 March 2016, https://doi.org/10.1186/ISRCTN43811467

## INTRODUCTION

Tobacco smoking is a major risk factor for developing pulmonary tuberculosis (TB)^1^, and the ‘dual epidemics’ of smoking and TB have led to a high burden of disease in South Asian countries^2-4^. TB patients who smoke have poorer treatment outcomes than non-smokers, manifesting as slower recovery from TB (OR=1.6; 95% CI: 1.0–2.1)^5^, higher rates of treatment failure (OR=2.3; 95% CI: 1.1–4.7)^6^ and higher risk of TB relapse (OR=3.1; 95% CI: 1.6–6.0)^7^ and death (OR=2.2; 95% CI: 1.3–3.7)^8^. Pakistan and Bangladesh are among eight countries that account for two-thirds of global TB cases^9,10^. In both countries, smoking prevalence is high for men (20% daily tobacco use in Pakistan and 35% in Bangladesh in 2015)^11,12^. Targeting smoking cessation through behavioral change interventions can be a successful strategy to increase quit attempts^13^. However, contextual factors extrinsic to the intervention that tend to hinder or bolster its effect^14^ including training and technical support to the providers, are likely to influence outcomes^15^.

TB diagnosed patients are typically requested to modify multiple behaviors, such as regularly taking medication, making dietary changes, adapting social behaviors (e.g. wearing a mask around others or not spitting on the ground), and potentially quitting smoking. Research on ‘multiple health behavior changes’ has gained traction^16-18^, yet whether these changes should be encouraged simultaneously, one at a time, or sequentially has not been conclusively determined^19,20^. Integrated strategies of delivering several behavioral change components at the same time have been found successful in cases of combined smoking cessation and physical activity^21-23^. In low-and middle-income countries, several trials have shown that interventions for multiple behaviors in participants with a pre-existing disease can be effectively integrated into routine care^24,25^. A previous trial involving offering behavioral counselling for smoking cessation to TB patients in Pakistan proved successful^26^. However, as time is restricted in TB clinics, whether such an integrated intervention can be successfully implemented by health workers requires further examination. A key concern is that additional behavioral change messages such as smoking cessation may crowd out TB management and containment messages.

Another important aspect of successful behavioral change relates to the way the intervention is delivered. If delivered as intended, interventions have the greatest likelihood of realizing the outcomes hypothesized to be produced^27,28^. However, in practice, behavioral change interventions are often delivered inconsistently^29^, resulting in increased variability in treatment outcomes^30,31^. To fully capture the extent to which different components of an intervention are delivered, intervention fidelity assessments are recommended within effectiveness studies^32,33^. Intervention fidelity refers to the degree to which an intervention is implemented as originally intended^34^, and both the content of the intervention and the quality with which it is delivered are important^35^. Measuring intervention fidelity enhances knowledge of the intervention components delivered and its application in real world settings^33^. Furthermore, understanding delivery of the features of the intervention encourages scientific replicability and optimizes intervention implementation in practice^32,36^.

Optimal delivery of an intervention is subject to multiple factors; the so-called ‘potential moderators’^35,37^. These include participant responsiveness, comprehensiveness of the intervention description, strategies to facilitate implementation, recruitment, and context^37^. Choosing a hospital as an intervention setting presents a context with special processes and microsystems^38,39^, which can create additional barriers to implementation. In routine TB care in Pakistan and Bangladesh – our two study countries – potential barriers for providing a smoking cessation intervention include social acceptability of smoking, high healthcare providers’ workload, priorities, motivation and capacity to deliver the intervention, as well as a lack of training in providing support to TB patients^40,41^. Facilitators for intervention delivery by healthcare providers include vocational motivation to provide support to patients, knowledge of health risks from smoking, and communication and rapport-building skills among health workers^42^. Training healthcare providers to deliver an additional behavioral change intervention within routine care is a promising approach considering pre-established trust, authority, access to the intervention recipients and knowledge of the additional behavioral changes required to best deal with the health condition^43,44^. On the other hand, healthcare providers may not be experts in behavior change techniques (BCT), may have limited time and could view the added tasks as minor, or as a distraction to their main task of providing care^38,45^. Few studies have assessed the effect of healthcare provider training on the delivery of different BCTs^18^.

Implementation of a standardized intervention across settings and countries can vary. Evaluating these highly contextual determinants can produce insights into the applicability and implementation of the intervention^46^. This study examines the fidelity to delivery of a novel multiple behavior change intervention addressing both TB and smoking-related behaviors in different contexts. It is unclear whether fidelity to delivery for a multiple behavior change intervention in routine practice can be achieved among health workers in the high stress and time scarce environment of TB clinics in Pakistan or Bangladesh. We explore whether TB care providers can deliver smoking cessation messages without compromising key TB-related messages such as medication adherence. We examine the differences in fidelity of delivery and prioritization of certain messages across the countries and public/private settings. Addressing these knowledge gaps is important to facilitate consistent provision of smoking cessation to patients with TB in contexts with high burdens of lung disease. Therefore, to better understand the role of providers and settings in the implementation of integrated smoking cessation support, our study aimed to achieve the following objectives:

1. To measure healthcare providers’ fidelity to an integrated tobacco cessation intervention delivered in routine TB care.
2. To describe differences in delivery of key behavioral support messages between Bangladesh and Pakistan, and between public and private TB care settings.

## METHODS

### Study design

A cross-sectional study for fidelity measurement was nested within a randomized controlled trial – the TB & Tobacco trial – investigating the effectiveness of cytisine (a smoking cessation pharmacotherapy) when added to BS for achieving smoking abstinence among TB patients^47^. The BS intervention for smoking cessation was developed and implemented in routine TB care in Bangladesh and Pakistan^42^. Full details of the trial and of the BS intervention are described elsewhere^42,48^.

### Setting

A purposive sample of health centers offering TB services in Bangladesh and Pakistan was enrolled (in the trial) in consultation with the respective National TB Control Programmes (NTP). Health centers were selected on the basis that they were functioning, designated TB diagnostic centers approved by NTP. Out of 32 health centers (17 subdistrict hospitals in Bangladesh and 15 secondary care hospitals in Pakistan) recruited across both urban and rural areas for the trial, 25 centers recorded and reported data for the fidelity measurement.

Pakistan has a plural health system. Therefore, when scaling up health interventions, the private healthcare settings cannot be ignored. Private sector engagement for smoking cessation, however, remains an important challenge because of the heterogeneous services ranging from formal to informal setups spread out in the country. The private sector is playing a significant part in health service provision in Pakistan, for example, 85% of TB patients initiate care seeking in the private setting^49,50^. It would be a missed opportunity by not integrating preventative services within these settings.

### Participants

Participants were TB providers from the sites participating in the TB & Tobacco trial. Any DOTS provider (including health workers, facilitators, or counsellors) involved in the management of TB treatment and care for the patients, were eligible for inclusion in this study.

### Sampling methods

A convenience sampling approach was taken for audio-recording sessions between June 2017 and April 2018. All providers participating in the TB & Tobacco study were asked to audio record 3–5 BS sessions for the fidelity assessment. Providers self-selected if they recorded as well as the day(s) and session(s) for their intervention delivery. Written informed consent was obtained from providers delivering the BS intervention at all trial sites for audio-recording a selection of their sessions for fidelity measurement. All recordings sent back to the study team that were audible on tape were included in the assessment.

TB patients receiving BS intervention, whose sessions were included in the study were requested to provide consent verbally prior to the recording (opt-in) and study information sheets were handed out after the session for patients to sign. Patients were given the option to withdraw consent for up to six days post-intervention. Ethics approval was obtained from the University of York Health Sciences Research Governance Committee (Ref: HSRGC/2016/144/B) and local ethics committees from Bangladesh (Ref: BMRC/NREC/2016–2019/1475) and Pakistan (Ref: no. 4–87/16/NBC-200 Part-B/RDC/4197). The datasets used and/or analyzed during the current study are available from the corresponding author on reasonable request.

### Intervention description

We developed a BS intervention guided by the design of prior tobacco cessation interventions in Pakistan^26^ and Nepal^51^. Key messages on the management of TB and smoking cessation were delivered to patients through flipbooks, leaflets and posters based on BCTs. The selection of BCTs for inclusion in our smoking counselling intervention were informed by focus group discussions as well as interviews with TB health workers, NTP staff and national tobacco control experts in each country^42^. The TB messages included in the intervention were derived from the counselling provided as part of the TB DOTS initiative. These messages were a part of standard practice and regularly delivered by the health workers. Health workers received training on delivering the intervention with a special focus on rapport-building with their patients. Training lasted 2 days on average, brought health workers from several clinics together, and included informational sessions and active role-play for different counselling scenarios. The intervention duration was about 15–20 min. Full details of the BS intervention development process, materials and reports can be accessed at: https://tbandtobacco.org/.

A health worker guide was given to all participants, covering the main topics:

- How to deliver the flipbook and leaflet and encourage patient engagement.
- Advice on following the TB medication schedule and seeking support from family and friends during TB treatment.
- Evidence for TB and tobacco interaction and health risks from smoked and smokeless tobacco.
- Information on nicotine dependency and its role in quit success.
- Advice for active listening and rapport building with different kinds of patients.
- Advice on how to follow up with patients on their tobacco cessation journey^42^.

### Fidelity measure

Index development was guided by the design of two previously applied indices for smoking cessation among patients with TB in Pakistan^52^ and smokeless tobacco users in Pakistan and the UK^53^, which were adapted for the purpose of this assessment. The method employed for quantifying the implementation of each intervention component and for developing the rating scales, in the fidelity index, has been validated in previous studies and found reliable (Krippendorff ’s α=0.80, and 0.66) in measuring intervention fidelity^52^. The BCT taxonomy^54^ was used as the coding framework for specifying the intervention components for both smoking cessation and TB care messages. The index represented two domains: adherence to delivery of content-based activities of the BS intervention (25 items; 12 TB related and 13 smoking cessation related) and quality of interaction-based activities (6 items). Each item was coded on a 3-point Likert scale (0=not implemented, 1=partially implemented, and 2=fully implemented) (Table 1). Definitions of partial implementation were provided for each item. A coding training session was developed, and bilingual coders (Urdu and English, or Bangla and English) were trained by one researcher via online sessions, following the training guidelines for the previous successfully conducted fidelity studies^52^. The observer at the private clinic in Pakistan was trained by a project researcher who had also conducted the training in the public clinics. Audio-recordings of the patient-provider BS sessions were administered by the site researchers. The recordings contained no personal identifying information.

**Table 1:** Average fidelity scores by providers/health centers

*Health center ID*	*Number of patients*	*Adherence score (content-based items)*	*Quality score (interactionbased items)*
		*Mean (SD)*	*Mean (SD)*
41	2	23.5 (9.19)	5.5 (0.71)
43	3	23.3 (3.21)	2.3 (1.53)
44	3	17.0 (2.00)	3.3 (0.58)
45	3	12.7 (3.06)	3.0 (0.00)
46	3	16.0 (1.73)	5.3 (2.08)
48	3	10.3 (7.57)	5.3 (4.16)
50	3	25.3 (4.04)	5.3 (2.08)
51	3	15.3 (7.02)	4.0 (0.00)
52	3	7.3 (1.15)	2.7 (0.58)
53	1	34.0 (-)	8.0 (-)
54	1	5.0 (-)	3.0 (-)
55	2	9.5 (0.71)	1.5 (2.12)
40	3	14.7 (0.58)	6.3 (1.53)
56	3	13.7 (1.53)	2.0 (1.73)
57	1	13.0 (-)	1.0 (-)
23	5	4.8 (1.64)	0.8 (0.84)
21	5	5.4 (1.67)	0.0 (0.00)
13	5	24.8 (4.44)	1.6 (2.51)
14	4	29.5 (2.08)	1.0 (0.82)
19	5	26.4 (9.61)	1.8 (1.64)
12	5	5.2 (1.92)	0.8 (0.84)
20	4	15.5 (8.74)	4.3 (2.87)
11	5	20.8 (2.39)	0.2 (0.45)
16	5	18.8 (8.17)	1.2 (1.10)
25	4	18.3 (11.32)	0.5 (0.58)
26	6	2.2 (1.33)	0.0 (0.00)
Indus hospital	6	22.7 (4.63)	2.50 (0.55)

Total score for the adherence scale, summarized by provider. Given is the average of the number of sessions for a provider, both for the Adherence score and the Quality score.

### Statistical analysis

No formal sample size calculation was conducted, as the planned analysis was descriptive in nature. In Bangladesh, two coders independently coded each audio-recording and discussed differences until a decision for a final score was made. In Pakistan, two coders independently coded each audio-recording and a third researcher independently coded a selected sample of audio-recordings for consistency checking and validation of the coding process. The subsample from the private hospital was coded by one researcher directly observing the BS sessions.

The method used for generating individual items of the index and developing corresponding rating scales (fully, partially, or not implemented) was adapted from previously validated fidelity indices^52,53^. However, because some items in this study included BCTs other than those for smoking cessation, it was important to test the reliability of the adapted fidelity index. Index scores were summarized for each item using mean, median and standard deviation, and for each domain of the index, i.e. adherence (to delivery of content-based activities) and quality (of interaction-based activities), using mean and standard deviation by provider. Inter-coder reliability of the item scores was computed using Krippendorff’s alpha, which provides a more conservative interpretation of the α coefficient than the conventional tests (poor for α<0.67, moderate 0.67–0.80, and good for α>0.80)^53,54^. All analyses were conducted in SAS version 9.4 (Cary, NC, USA)^55^.

Fidelity to delivery of the intervention was presented as the proportion of patient-provider sessions that fully or partially implemented BS messages, or did not implement them.

## RESULTS

Ninety-six audio-recordings of intervention sessions, 37 from 15 public hospital sites in Bangladesh and 59 from 10 sites in Pakistan (9 public and 1 private hospital) were included in the descriptive analysis.

### Description of item and domain scores and inter-coder reliability of the fidelity index

The average fidelity scores for both domains across sessions (adherence and quality – maximum scores possible were 56 and 10, respectively) are described in Table 1. Mean adherence scores ranged between 2.2 (SD=1.33) and 29.5 (2.08) and mean quality scores ranged between 0.0 and 6.3 (1.53).

The overall mean Krippendorff’s α for individual items was 0.548 for adherence to content and 0.357 for quality of interaction, which indicates poor inter-coder reliability overall (Table 2). Items 3, 6–8, 12–14 and 29–31 showed very little variance (Supplementary file Table S1). Items 3, 8, 29–31 were found to have a floor effect, which means values close to the lowest score. Providers did not implement these elements of BS in the majority of sessions.

**Table 2:** Item description, mean coder scores for items across sessions, and inter-coder reliability estimates

*Item number*	*Item content*	*Pakistan*			*Bangladesh*		
		*Coder 1 Mean score*	*Coder 2 Mean score*	*Krippendorff’s α (95% CI)*	*Coder 1 Mean score*	*Coder 2 Mean score*	*Krippendorff’s α (95% CI)*
**Adherence (content-based items)**							
**Content items TB medication adherence**							
1	Provide information about health consequences: You can be cured of TB with medication	0.67	0.73	0.956 (0.868–1.00)	1.51	1.30	**0.617 (0.361–0.872)**
2	Advise or agree on how to perform behavior: Keep taking medicines regularly	1.53	1.53	0.943 (0.831–1.00)	1.78	1.62	**0.473 (-0.053–0.895)**
3	Advise or agree on how to perform behavior: Never take a double dose	0.12	0.07	0.791 (0.791–0.374)	0.03	0.00	**0.000 (-1.00–0.000)**
4	Advise or agree on how to perform behavior: Keep taking medicine even if they have positive or negative effects	0.27	0.33	0.852 (0.630–1.00)	0.38	0.22	0.684 (0.263–1.00)
**Content items lifestyle**							
5	Advise on, arrange or provide practical help: Get help from friends/family not to forget medicine	0.75	0.80	0.957 (0.871–1.00)	0.27	0.22	0.875 (0.626–1.00)
6	Advise or agree on how to perform behavior: Keep coming to scheduled appointments	0.41	0.38	0.870 (0.717–0.989)	0.19	0.22	0.734 (0.393–0.992)
7	Advise on, arrange, or provide emotional social support: Having TB is not shameful	0.76	0.85	0.913 (0.781–1.00)	0.05	0.22	**0.365 (-0.270–1.00)**
8	Advise on, arrange, or provide emotional social support: You will need support of family and friends to get better	0.04	0.18	**0.484 (-0.299–1.00)**	0.11	0.11	**0.479 (-0.304–1.00)**
9	Advise or agree on how to perform the behavior: Encourage the patient to adopt a healthy lifestyle	0.96	0.98	0.844 (0.716–0.969)	0.43	0.38	**0.580 (0.283–0.847)**
10	Advise or agree on how to perform the behavior: Abstain from all tobacco products	0.80	0.80	0.994 (0.981–1.00)	0.27	0.05	**-0.074 (-0.968–0.642)**
11	Advise or agree on how to perform the behavior: Healthy and nutritious food	1.41	1.45	1.00 (1.00–1.00)	0.86	0.86	0.674 (0.403–0.891)
12	Advise or agree on how to perform the behavior: Getting lots of rest	0.67	0.65	0.952 (0.855–1.00)	0.11	0.11	**0.479 (-0.304–1.00)**
13	Advise or agree on how to perform the behavior: Getting lots of fresh air	0.78	0.87	0.958 (0.874–1.00)	0.05	0.00	**0.000 (-1.00–0.000)**
**Content items tobacco cessation**							
14	Advise or agree on how to perform the behavior: Abstaining from alcohol and tobacco	0.49	0.65	0.858 (0.740–0.949)	1.05	0.92	**0.371 (-0.079–0.732)**
15	Assess current and past smoking behavior: Check if patient uses tobacco	0.71	0.65	1.00 (1.00–1.00)	1.46	1.24	**0.513 (0.209–0.979)**
16	Provide information about health consequences of performing the behavior: Positive and negative effects on TB of quitting tobacco	0.73	0.78	0.974 (0.939–1.00)	1.11	1.19	0.783 (0.659–0.907)
17	Use methods to emphasize consequences: Positive effects of quitting, negative effects of not quitting	0.63	0.62	0.855 (0.661–1.00)	1.57	1.62	**0.423 (0.010–0.753)**
18	Provide information about health consequences of performing the behavior: Quitting improves health and saves money	0.24	0.33	0.766 (0.455–1.00)	0.59	0.49	0.865 (0.662–1.00)
19	Advise on how to avoid exposure: Gradual cessation is not effective	0.43	0.53	0.894 (0.716–1.00)	0.35	0.32	**0.587 (0.232–0.892)**
20	Advise on how to avoid exposure: Abrupt cessation	0.39	0.38	0.796 (0.583–0.948)	0.43	0.30	**0.598 (0.255–0.886)**
21	Explain why it is better to stop abruptly: Abrupt cessation	0.12	0.18	**0.543 (-0.066–1.00)**	0.32	0.27	**0.473 (0.052–0.895)**
22	Set a quit date: Ask the patient whether they want to quit/set a quit date	0.04	0.04	**-0.010 (-1.00–0.495)**	0.54	0.49	**0.651 (0.371–0.930)**
23	Set a quit date: Together with the patient suggest to find a quit date	0.04	0.04	1.00 (1.00–1.00)	0.46	0.51	0.723 (0.459–0.925)
24	Provide information about health consequences: Withdrawal symptoms might occur	0.98	0.98	1.00 (1.00–1.00)	0.57	0.54	0.924 (0.819–1.00)
25	Provide information about health consequences: Describe withdrawal symptoms and how to handle them	0.90	0.91	0.972 (0.931–1.00)	0.59	0.70	0.907 (0.817–0.974)
**Quality (interaction-based items)**							
26	Build general rapport	0.75	0.69	0.900 (0.749–1.00)	1.08	0.73	**0.232 (-0.174–0.541)**
27	Elicit and answer questions	0.22	0.25	0.733 (0.393–1.00)	0.95	1.24	**0.167 (-0.350–0.588)**
28	Provide reassurance	0.10	0.18	0.838 (0.513–1.00)	0.84	0.95	**0.582 (0.307–0.800)**
29	Use reflective listening	0.02	0.07	**-0.010 (-1.00–0.505)**	0.35	0.30	**0.059 (-0.417–0.468)**
30	Offer/direct towards written materials	0.00	0.00	**0.000 (0.000–0.000)**	0.11	0.38	**0.376 (-0.123–0.875)**
31	Tailor interactions appropriately	0.00	0.00	**0.000 (0.000–0.000)**	0.32	0.38	0.724 (0.356–1.00)

Mean Krippendorff’s alpha for Adherence was 0.548 and for Quality was 0.357 for Bangladesh; for Adherence it was 0.846 and for Quality it was 0.410 for Pakistan. The anchors for 13 items were 0 (not implemented), 1 (partially implemented), 2 (fully implemented), and for 18 items were 0 or 2. Items in bold show low agreement (i.e. α<0.67).

### Adherence to delivery of intervention content and quality of interaction

For Bangladesh, BS messages on TB medication and compliance were implemented better than the smoking cessation messages, in general (Figure 2). ‘Importance of medicine for cure’ and ‘taking it regularly’ were implemented in 91.9% (95% CI: 78.7–97.2) and 70.3% (95% CI: 54.2–82.5), respectively. ‘Emphasizing the positive effects of quitting’ was implemented fully in 83.7% (95% CI: 68.9–92.4) of the sessions. ‘Abstaining from alcohol and tobacco’ was advised in almost all of the sessions, but this advice was marked as partial. To qualify for full implementation, both alcohol and tobacco would have to be addressed in this item, whereas in our sample alcohol was rarely discussed. Patients were asked whether they used tobacco in 70.3% (95% CI: 54.2–82.5) of sessions, yet a quit date was only set in 32.4% (95% CI: 19.6–48.5) of sessions.

**Figure 1:**
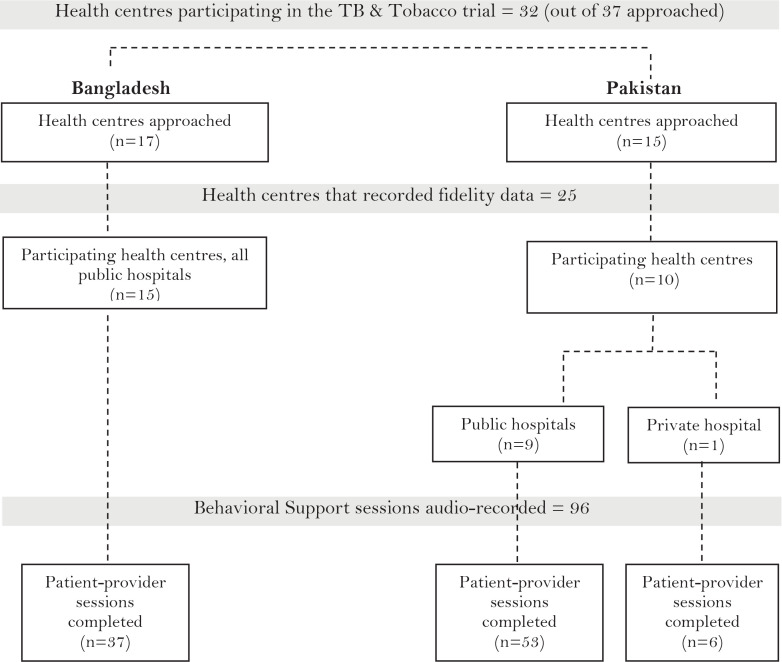
Study flow chart

**Figure 2:**
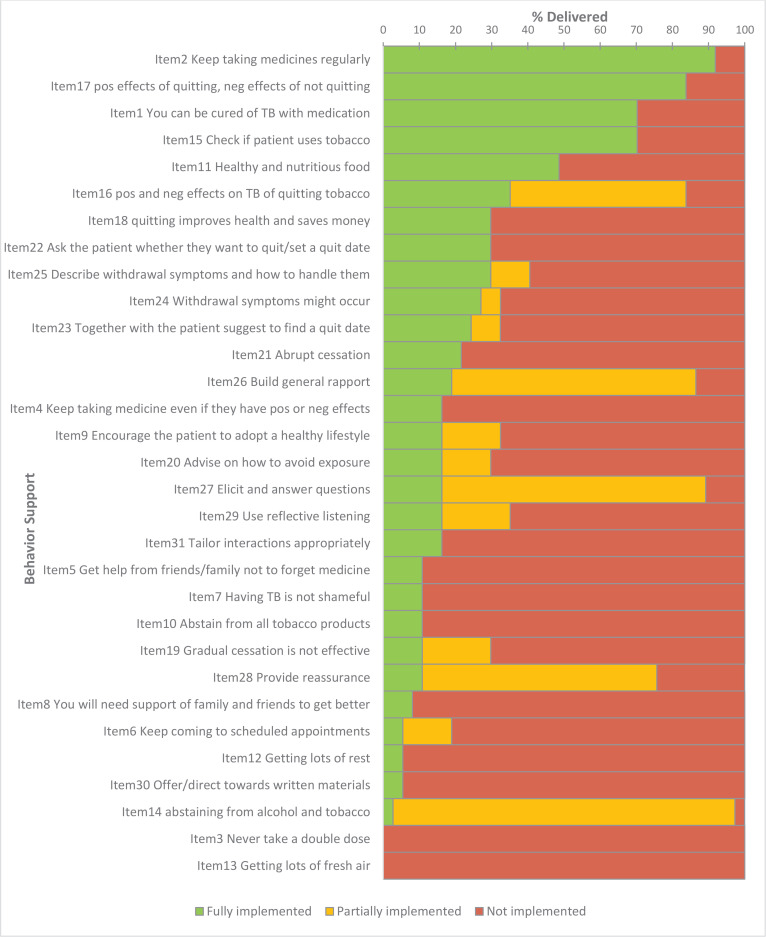
Behavior Support intervention fidelity (Bangladesh)

In the quality of interaction items, building general rapport (86.5%, 95% CI: 72.0–94.1), eliciting and answering questions (89.2%, 95% CI: 75.3–95.7) and providing reassurance (75.7%, 95% CI: 59.9–86.6) were implemented well. Contextual tailoring, for instance, changing the examples of which foods to eat depending on patients’ socioeconomic status, occurred in only 16.2% (95% CI: 7.7–31.1) of sessions.

In Bangladesh, messages consistently not implemented were ‘to never take a double dose of TB medicine’, ‘to get fresh air as part of a healthy lifestyle’, and ‘to refer to the leaflet for further information on smoking cessation’.

**Figure 3:**
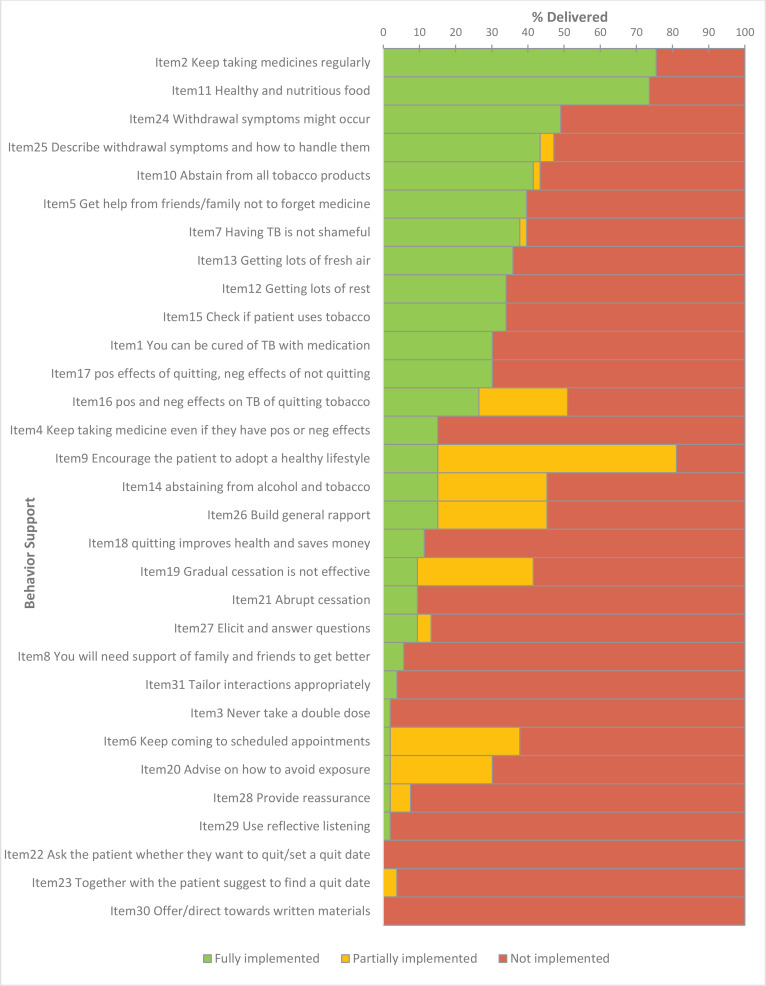
Behavior Support intervention fidelity (Pakistan - Public)

In Pakistan, in the National TB control programme – (public settings), BS elements relating to TB compliance were implemented in 75.5% (95% CI: 62.4–85.1) of the sessions (Figure 3). Nutrition advice was implemented in 73.6% (95% CI: 60.4–83.1) of the sessions. Advice on getting help from friends or family to not forget medicine was implemented in 39.6% (95% CI: 27.6–53.1). Advice on abstaining from all tobacco products was implemented in about half of the sessions (43.4%, 95% CI: 31.0–56.7). Tobacco use status was asked about in 34.0% (95% CI: 22.7–47.4) of sessions. Information about withdrawal symptoms (49.1%, 95% CI: 36.1–62.1) and how to handle them (47.1%, 95% CI: 34.4–60.3) was implemented in about half of the sessions.

In the quality of interaction items, building general rapport was implemented in 45.3% (95% CI: 32.7– 58.6). Implementation was low for the rest of the quality items.

**Figure 4:**
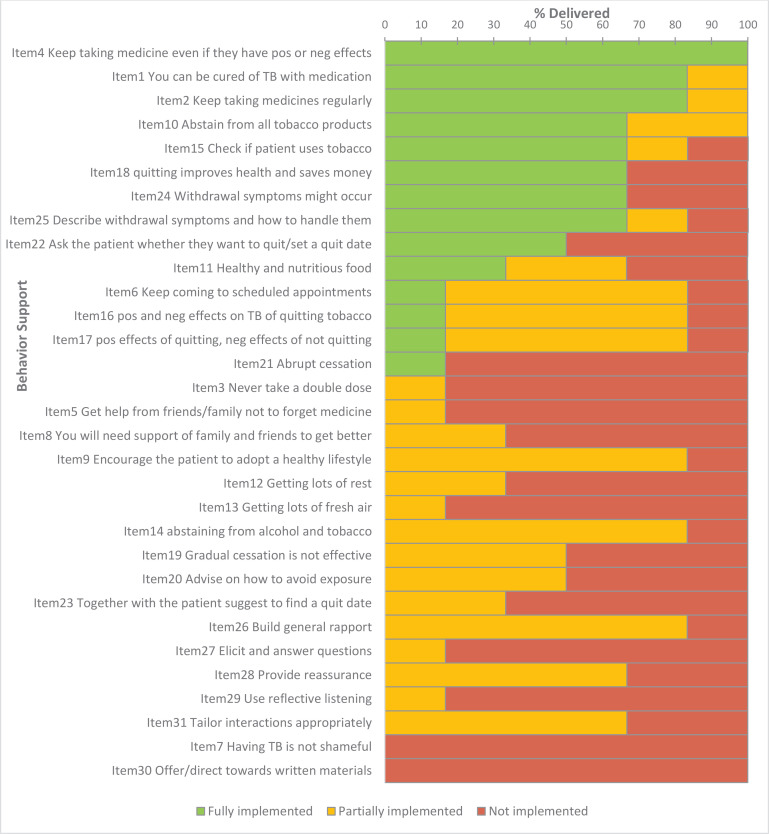
Behavior Support intervention fidelity (Pakistan - Private)

In the private clinic in Pakistan, messages relating to TB compliance were implemented in 83.3% (95% CI: 43.7–97.0) of the sessions (Figure 4). Checking tobacco use status, advising on quitting, and providing information on withdrawal symptoms were all implemented in 66.7%, (95% CI: 30.0–90.3) and advice on management of withdrawal symptoms was implemented in 83.3% (95% CI: 43.7–97.1) of the sessions. Asking the patient whether they wanted to quit was implemented in 50.0% (95% CI: 18.8–81.2) of the sessions, whilst setting a quit date with the patient was only partially implemented in 33.3% (95% CI: 9.6–70.0) of the sessions. Advice on getting family support for both TB medication and smoking cessation was only partially implemented in a few cases. Messages on adopting a healthier lifestyle 83.3% (95% CI: 43.7–97.1), getting lots of rest 33.3% (95% CI: 9.7–70.0) and getting fresh air 16.7% (95% CI: 3.0–56.4) were only implemented partially.

In the quality of interaction items, building rapport was partially implemented in 83.3% (95% CI: 43.7– 97.1) of the sessions. Providing reassurance and tailoring interactions was partially implemented in 66.7% (95% CI: 30.0–90.3) of the sessions; implementation was negligible for the rest of the items.

## DISCUSSION

Our findings show that most patients received counselling on TB medication adherence and on the positive effects of quitting smoking. A healthy lifestyle and methods to manage withdrawal symptoms were explained, and patients’ smoking status was assessed. TB-related messages were consistently delivered more proficiently than the smoking cessation-related messages. Below we discuss our findings in relation to the application of the fidelity index and delivery of the intervention, including differences between providers and settings.

Fidelity to behavioral support messages was high for the majority of main messages. Fidelity was less than anticipated for quality of interaction items, discussing a quit date, and handing out a leaflet during the session. While provision of the leaflet was not audibly recorded in these sessions, it is possible that the leaflets were handed out after the recording had ended or were available for patients to take at their leisure. These findings concur with other smoking cessation studies reporting fidelity to delivery of intervention activities. For example, setting a quit date and offering information leaflets were also found to be least implemented in our previous research, which might be due to the nature of the activity or exchange of information not being captured during the audio-recording of the session^52^. It appears that setting a quit date was infrequently a component of the recorded counselling sessions in the current study. This could be a problem as higher quality of goal setting has been linked to increased quit success^56^. On the other hand, patients returned frequently to the clinic to receive TB medication^57^, granting the health workers multiple opportunities to raise the topic of smoking after the initial consultation. It is therefore possible that the process of finding a quit date occurs less frequently during the initial counselling session and more implicitly in the course of the provider-patient relationship. In a scoping review on the reporting of intervention fidelity in tobacco treatment studies, very few studies reported on nonspecific treatment effects such as the quality of interaction^58^. Whether this is due to poor implementation or a lack of measurement and reporting of the quality of interaction is not yet known.

The process evaluation of the TB & Tobacco trial found that time constraints and high patient volumes frequently forced health workers to adapt the intervention to their needs, by excluding the flipbook or by focusing on TB messages and on statements about health risks from smoking^41^. Such adaptations can only be captured as absences by the fidelity index. Future iterations might include a second coding round for emerging constructs, noting which additional or different contents were delivered by health workers and mapping those to the BCTs. Our sample of audio-recordings was collected over a period of time to account for fidelity changes over time as providers’ proficiency increased. However, the final sample per site was too limited (3–5 recordings) to draw robust conclusions about individual health worker proficiency developments. Future studies should ensure a larger sample per site to account for temporal changes.

Another aspect of the fidelity index that can be critically assessed is the lack of weighting of individual messages. As the intervention was brief, at 15–20 minutes with 8 pages in the flipbook, all messaging was already considered essential. The quality items could potentially be further refined and examined for their importance in intervention delivery^18^. For instance, in health systems with much authority resting on the practitioner^59^, a reflective listening approach with room for patient questions may not be as well received as in systems more focused on shared decision-making. Our index differentiated between several types of quality factors as recommended^32,33^, and future versions of the index could consider assigning a weighting approach to individual items.

The trained health workers were able to deliver multiple behavior change techniques in the same intervention, yet the messages on TB medication adherence and TB-related lifestyle changes were provided more proficiently. One likely reason is that health workers were reasonably familiar with those messages from their daily work, whereas the smoking cessation advice was new to them. Sequentially, the intervention moved from TB advice to smoking cessation advice, which is in line with the flow of the TB counselling as part of DOTS and is theory-based in moving from general lifestyle advice to specific smoking cessation advice^42^. It is possible that the counselling structure was not always followed exactly as planned, particularly under time constraints in a busy clinic^60^. We found no evidence for smoking message delivery compromising medication adherence messages, which indicates that multiple behavioral changes can be advocated for, using our intervention.

Differences between Pakistan and Bangladesh were found in the quality of interaction items and in the frequency of setting a quit date. In the Pakistan sample, eliciting and answering questions took place less frequently than in Bangladesh. One possible explanation for these low quality of interaction scores in Pakistan could be the limited time available for each counselling session. Active listening, posing open questions and opening up space for patients to talk about their experiences takes time, whereas patients may have been waiting for a diagnosis and medication for several hours and eager to get back home or to work^41^. The health workers also had a higher workload in some of the tertiary care hospitals in Pakistan, consisting of the administrative work of registering new patients, delivering medication, and providing TB counselling^61^, which can act as a barrier to service provision^62^. They may pragmatically have opted for a didactic interaction rather than a two-way conversation in order to allow them to see all patients waiting within the allotted time. In the private setting in Pakistan, setting a quit date was addressed more frequently than in the public sector (33.3% vs 3.8% of sessions), as was checking for tobacco use. As the private setting included only one clinic, this could be a site-specific effect illustrating high provider motivation, or an artefact of the observation. The concept of setting a quit date together with patients still needs to be explored further with health workers to better understand why this component of the intervention was less frequently addressed.

### Limitations

There are several limitations in our study. First, potential moderating factors in providers and also in patients were not taken into account in this assessment. Individual characteristics of the providers and patients (e.g. gender, age) may have an effect on intervention delivery and quality of interaction^63^, and providers who smoke themselves may be less likely to deliver cessation advice^64,65^. As the intervention was provided to all newly diagnosed TB patients entering care, it is possible that those whose sessions were recorded were non-smokers. In that case, providers may have received that information in a different way before starting the counselling and their lack of addressing smoking status or setting a quit date might be their tailoring of the intervention to patient needs. The fidelity assessment may need to be complemented with additional data sources outside the main intervention session to account for such deviations. Second, private settings are not well represented in this sample as only one such clinic is included. This should be considered when interpreting the findings of this study in relation to the private healthcare context. Finally, sample selection for audio-recording was left at the discretion of the providers for flexibility, as patient load can be heavy at certain times in the outpatient department of public hospitals. The trial researchers based at each hospital could have audio-recorded these patient-provider sessions, which might have bypassed the self-selection bias but due to patient privacy it was not considered. Moreover, presence of an observer may adversely affect patient-provider rapport building in the intervention sessions and it was considered less obtrusive for the providers to record their own sessions rather than the presence of researchers in the room to facilitate the process.

### Research implications

The fidelity index provides a useful method to capture and quantify individual behavioral change items, enabling better description of intervention activities that were actually delivered during practice. Future assessments of multiple behavioral change interventions should consider more than one data source for fidelity assessment, to allow for a complete understanding of the specific context of an intervention session. The balance between fidelity and adaptation may be an important indicator of implementation success^66^. A larger sample for fidelity measurement at each site over a longer period would enable researchers to also assess temporal variations in delivery, and more information about providers and recipients of the intervention would be useful to better judge adaptations made by health workers.

### Practice implications

Our fidelity findings indicate that the TB & Tobacco behavioral support intervention was implemented successfully, and cessation messages were integrated into the TB adherence counselling. A future iteration of the intervention should consider additional training on patient rapport-building for health workers. To ensure integrity of the intervention, health workers could be encouraged to deliver a modified, core message programme instead of self-selecting messages, when pressed for time. Policymakers and TB managers could support the inclusion of an integrated tobacco cessation intervention into routine care by providing additional resources to separate counselling from TB administrative tasks.

## CONCLUSIONS

A smoking cessation behavior support programme based on behavior change technique-guided counselling and integrated into a TB behavior counselling session was examined. Fidelity to the intervention delivery was found to be high for TB-related messages but not for the main smoking cessation messages. Clinic contexts may play a mediating role in health workers’ opportunities to deliver the intervention as planned.
