# Supplementary material for: Measuring fidelity to delivery of a new smoking cessation intervention integrated into routine tuberculosis care in Pakistan and Bangladesh: Contextual differences and opportunities
Source: Tob Induc Dis. 2021 Apr 8;19:24. doi: 10.18332/tid/133054 (PMC8029647; doi:10.18332/tid/133054)
Supplement: Supplementary file 1 [file TID-19-24-s1.pdf]

Supplementary Table S1

Descriptive statistics for item scores

ITEM	Coder PK1 Score			Coder PK2 Score			Coder BD1 Score			Coder BD2 Score		
	Mean	Median	*SD	Mean	Median	SD	Mean	Median	SD	Mean	Median	SD
**ADHERENCE (content-based items)**												
Item1	0.67	0.00	0.95	0.73	0.00	0.97	1.51	2.00	0.87	1.30	2.00	0.97
Item2	1.53	2.00	0.86	1.53	2.00	0.86	1.78	2.00	0.63	1.62	2.00	0.79
Item3	0.12	0.00	0.48	0.07	0.00	0.38	0.03	0.00	0.16	0.00	0.00	0.00
Item4	0.27	0.00	0.70	0.33	0.00	0.75	0.38	0.00	0.79	0.22	0.00	0.63
Item5	0.75	0.00	0.98	0.80	0.00	0.99	0.27	0.00	0.69	0.22	0.00	0.63
Item6	0.41	0.00	0.57	0.38	0.00	0.56	0.19	0.00	0.40	0.22	0.00	0.53
Item7	0.76	0.00	0.97	0.85	0.00	0.99	0.05	0.00	0.33	0.22	0.00	0.63
Item8	0.04	0.00	0.28	0.18	0.00	0.58	0.11	0.00	0.46	0.11	0.00	0.46
Item9	0.96	1.00	0.53	0.98	1.00	0.62	0.43	0.00	0.73	0.38	0.00	0.76
Item10	0.80	0.00	0.98	0.80	0.00	0.97	0.27	0.00	0.69	0.05	0.00	0.33
Item11	1.41	2.00	0.92	1.45	2.00	0.90	0.86	0.00	1.00	0.86	0.00	1.00
Item12	0.67	0.00	0.95	0.65	0.00	0.95	0.11	0.00	0.46	0.11	0.00	0.46
Item13	0.78	0.00	0.99	0.87	0.00	1.00	0.05	0.00	0.33	0.00	0.00	0.00
Item14	0.49	0.00	0.67	0.65	0.00	0.75	1.05	1.00	0.33	0.92	1.00	0.43
Item15	0.71	0.00	0.97	0.65	0.00	0.95	1.46	2.00	0.90	1.24	2.00	0.98
Item16	0.73	0.00	0.83	0.78	1.00	0.85	1.11	1.00	0.74	1.19	1.00	0.70
Item17	0.63	0.00	0.94	0.62	0.00	0.93	1.57	2.00	0.83	1.62	2.00	0.79
Item18	0.24	0.00	0.65	0.33	0.00	0.75	0.59	0.00	0.93	0.49	0.00	0.87
Item19	0.43	0.00	0.61	0.53	0.00	0.66	0.35	0.00	0.63	0.32	0.00	0.63
Item20	0.39	0.00	0.70	0.38	0.00	0.62	0.43	0.00	0.73	0.30	0.00	0.66
Item21	0.12	0.00	0.48	0.18	0.00	0.58	0.32	0.00	0.75	0.27	0.00	0.69
Item22	0.04	0.00	0.28	0.04	0.00	0.27	0.54	0.00	0.90	0.49	0.00	0.87
Item23	0.04	0.00	0.20	0.04	0.00	0.19	0.46	0.00	0.84	0.51	0.00	0.80
Item24	0.98	0.00	1.01	0.98	0.00	1.01	0.57	0.00	0.83	0.54	0.00	0.87
Item25	0.90	0.00	0.98	0.91	0.00	0.99	0.59	0.00	0.80	0.70	0.00	0.91

QUALITY (interaction-based items)											
Item26	0.75	1.00	0.82	0.69	0.00	0.84	1.08	0.55	1.00	0.73	1.00	0.56
Item27	0.22	0.00	0.58	0.25	0.00	0.64	0.95	0.47	1.00	1.24	1.00	0.60
Item28	0.10	0.00	0.41	0.18	0.00	0.55	0.84	0.60	1.00	0.95	1.00	0.74
Item29	0.02	0.00	0.14	0.07	0.00	0.38	0.35	0.63	0.00	0.30	0.00	0.70
Item30	0.00	0.00	0.00	0.00	0.00	0.00	0.11	0.46	0.00	0.38	0.00	0.79
Item31	0.00	0.00	0.00	0.00	0.00	0.00	0.32	0.75	0.00	0.38	0.00	0.79

*SD is standard deviation

Items highlighted in grey are those with SD < .50, showing little variance

The response option anchors for each item were 0 = not implemented, 1= partially implemented and 2= fully implemented; for items 1,2,3,4,5,7,8,10,11,12,13,15,16,17,22,24,30,31 the response option anchors were 0= not implemented and 2= implemented.

© 2021 Boeckmann M. et al.
